# Supplementary material for: MetaRibo-Seq measures translation in microbiomes
Source: Nat Commun. 2020 Jun 29;11:3268. doi: 10.1038/s41467-020-17081-z (PMC7324362; doi:10.1038/s41467-020-17081-z)
Supplement: Supplementary file 10 — Supplementary Data 7 [file 41467_2020_17081_MOESM10_ESM.zip › File2/Confidence_High_Taxonomy/42518_out.krona.html]

Javascript must be enabled to view this page.

members
magnitude
magnitudeUnassigned
count
unassigned
taxon
rank

42518\_out

3

2
3
superkingdom

phylum
1
201174

1760
class
1

1
species

SRS049900\_contig\_number\_contig-100\_22106.79804
1883427

2
phylum
1239

186801
class
2

2
order
186802

186803
family
1

572511
1
genus

SRS053214\_contig\_number\_contig-100\_39334.39335

family
1
216572

genus
1
459786

1262910
1
species

SRS100021\_contig\_number\_11195
